# LncRNA *GACAT3*: A Promising Biomarker and Therapeutic Target in Human Cancers

**DOI:** 10.3389/fcell.2022.785030

**Published:** 2022-01-20

**Authors:** Xin Yuan, Zihui Dong, Shen Shen

**Affiliations:** ^1^ Department of Infectious Diseases, The First Affiliated Hospital of Zhengzhou University, Zhengzhou, China; ^2^ Precision Medicine Center, Gene Hospital of Henan Province, The First Affifiliated Hospital of Zhengzhou University, Zhengzhou, China

**Keywords:** lncRNA, cancer, biomarker, GACAT3, molecular mechanisms

## Abstract

Long non-coding RNAs (lncRNAs) are a class of functional RNA molecules that do not encode proteins and are composed of more than 200 nucleotides. LncRNAs play important roles in epigenetic and gene expression regulation. The oncogenic lncRNA *GACAT3* was recently discovered to be dysregulated in many tumors. Aberrant expression of *GACAT3* contributes to clinical characteristics and regulates multiple oncogenic processes. The association of *GACAT3* with a variety of tumors makes it a promising biomarker for diagnosis, prognosis, and targeted therapy. In this review, we integrate the current understanding of the pathological features, biological functions, and molecular mechanisms of *GACAT3* in cancer. Additionally, we provide insight into the utility of *GACAT3* as an effective diagnostic and prognostic marker for specific tumors, which offers novel opportunities for targeted therapeutic intervention.

## Introduction

Despite the countless advances of modern medicine and technology, the incidence and mortality of cancer remain high with an estimated 18.1 million new cancer cases and 9.6 million cancer-related deaths worldwide in 2018 alone (Bray et al., 2018; Ferlay et al., 2019; Lim et al., 2019). The rising incidence of cancer reflects both the prevalence of the disease, as well as improved detection and diagnostic capabilities. In contrast, the high mortality of cancer patients underscores the progress that still needs to made, especially in advanced or recurrent cancers with poor prognoses that remain difficult to treat (Li et al., 2018b; Moody et al., 2020). Effectively combatting cancer involves detecting the malignancy as early as possible and developing novel targeted therapies to treat cancers at advanced or recurrent stages. Both of these aims would be greatly aided by the development of novel and reliable biomarkers.

High-throughput technologies, such as next-generation sequencing, have revealed a variety of non-protein coding transcripts or non-coding RNAs (ncRNAs). ncRNAs play important roles in a variety of cellular and physiological functions (Chen, 2016). The largest class of ncRNAs is long non-coding RNAs (lncRNAs), which are functional RNA molecules of more than 200 nucleotides and include intergenic transcripts, enhancer RNAs (eRNAs), and sense or antisense transcripts that overlap other genes (Kopp and Mendell, 2018). LncRNAs play important roles in epigenetic and gene expression regulation at both the transcriptional and post-transcriptional levels. LncRNAs have also been implicated in stem cell maintenance and differentiation, cell autophagy and apoptosis, and embryonic development (Qian et al., 2019). The expression of specific lncRNAs is altered in many cancers, suggesting that lncRNAs contribute to tumorigenesis and/or tumor cell function (Xia et al., 2019; Qi-Dong et al., 2020; Shen et al., 2020; Ren et al., 2021).

The lncRNA gastric cancer-associated transcript 3 (*GACAT3*, also known as AC130710) is one such example of a cancer-associated lncRNA (Li et al., 2020). *GACAT3* functions as a competing endogenous RNA (ceRNA) and is encoded by human chromosome 2p24.3 (Xia et al., 2014; Cao et al., 2020). Previous studies have shown that *GACAT3* is involved in non-malignant diseases, including osteoarthritis (Li et al., 2018a). More recently, *GACAT3* was reported to be abnormally expressed in many malignant tumors, including gastric cancer, hepatocellular carcinoma, colorectal cancer, breast cancer, bladder cancer, glioma, ovarian cancer, and non-small cell lung cancer (Klymenko et al., 2017). Accumulating evidence demonstrates that *GACAT3* participates in the tumorigenesis of multiple human cancers.

The molecular function of *GACAT3* is well-established and suggests that *GACAT3* is a promising prognostic marker and target for therapy in specific cancers. Here, we review *GACAT3* expression in human cancers, the association of *GACAT3* with clinical characteristics, and the biological functions and regulatory mechanisms of *GACAT3* in tumorigenesis.

## Expression of *GACAT3* in Cancer

Many studies show that *GACAT3* is dysregulated in cancers, such as gastric cancer, colorectal cancer, hepatocellular carcinoma, breast cancer, bladder cancer, and glioma. Overexpression of *GACAT3* correlates with clinicopathological features, including tumor size, tumor grade, stage of development, and lymph node metastasis. Furthermore, *in vitro* and *in vivo* experiments reveal that *GACAT3* enhances cellular proliferation, migration, invasion, and tumor growth, which all contribute to aggressive tumor progression. Below we review the expression of *GACAT3* in specific tumors, introduce the primary clinicopathological significance of *GACAT3* in these tumors, and discuss the common *GACAT3*-regulated biological processes. *GACAT3* expression and the relevant clinicopathological features of specific cancers are summarized in Table 1.

**TABLE 1:** *GACAT3* expression and association with clinicopathological features of human cancers.

Cancer type	Expression	Relevant clinical characteristics	References
Gastric cancer	Upregulated in tissues and MGC-803 cells; downregulated in BGC-823, SGC-7901, and AGS cells	Overall survival, tumor size, TNM stage, and distant metastasis	Lin et al. (2018), Xu et al. (2014), Feng et al. (2018)
Colorectal cancer	Upregulated	Tumor size, invasion depth, TNM stage, lymph node metastasis, and CA19-9 level	Ye et al. (2020), Zhou et al. (2018)
Hepatocellular carcinoma	Upregulated	Overall survival and disease-free survival	Li et al. (2020)
Breast cancer	Upregulated	Overall survival, preoperative MRI perfusion-related diffusion, and perfusion score	Hu et al., 2019a
Glioma	Upregulated	Overall survival	Pan et al. (2019)
Bladder cancer	Upregulated	High grade and stage	Zhang et al. (2021)
Non-small cell lung cancer	Upregulated	Radiotherapy sensitivity	Yang et al. (2018)

### Gastric Cancer

Based on the 2019 Global Cancer Statistics, gastric cancer (GC) remains one of the most common malignancies (De Re, 2018; Siegel et al., 2019; Smyth et al., 2020). Numerous studies have demonstrated that abnormal expression of lncRNAs is associated with the occurrence and development of GC (Song et al., 2013; Li et al., 2014; Li et al., 2016; Dragomir et al., 2020). *GACAT3* was first associated with GC by Chen *et al.* who officially named the lncRNA “gastric cancer associated transcript 3 (*GACAT3*)” (Chen et al., 2014; Sun et al., 2016). Since then, an accumulation of studies have confirmed that *GACAT3* is significantly upregulated in GC tissues (Xu et al., 2014; Fanelli et al., 2018; Lin et al., 2018). In follow-up cell line experiments, significantly higher expression of *GACAT3* was observed in MGC-803 cells compared to the normal gastric GES-1 cell line, whereas *GACAT3* was expressed at remarkably lower levels in BGC-823, SGC-7901, and AGS gastric cancer cells compared to GES-1 cells. This opposing response among cell lines has been attributed to the heterogeneity of GC tumors (Xu et al., 2014). Despite these opposing results in cell lines, high *GACAT3* expression in GC tissues is positively associated with tumor size, tumor-node-metastasis (TNM) stage, and distal metastasis (Xu et al., 2014). Functionally, siRNA-mediated knockdown of *GACAT3* results in inhibition of cell proliferation in HGC-27 cells. Similarly, suppression of cell growth, increased apoptosis, and drug resistance occur after knockdown of *GACAT3* in SGC-7901 and BGC-823 cells (Shen et al., 2016; Lin et al., 2018). *GACAT3* also influences colony formation, migration, and invasion in GC cells (Shen et al., 2016; Feng et al., 2018). In summary, although *GACAT3* expression varies among GC cancer cell lines, the overwhelming majority of evidence suggests that *GACAT3* functions as an oncogene in GC.

### Colorectal Cancer

Colorectal cancer (CRC) is the world’s fourth most fatal malignancy, accounting for almost 900,000 deaths per year (Dekker et al., 2019; Ferlay et al., 2019; Arnold et al., 2020). Over the next decade, CRC is estimated to account for more than 2.2 million new cases and 1.1 million deaths (Arnold et al., 2017). As the clinical manifestations of CRC are only evident at advanced stages, it is critically important to develop novel sensitive biomarkers to aid the early diagnosis of CRC, which would vastly improve patient survival and prognosis (Zhou et al., 2018; Tieng et al., 2020; Shamsuddin et al., 2021). *GACAT3* is a promising biomarker for CRC as it is overexpressed in CRC tissues and is upregulated in the CRC cell lines HT29, HCT116, SW480, and LoVo relative to the normal colorectal epithelial cell line FHC (Zhou et al., 2018; Ye et al., 2020). Functional studies demonstrate that siRNA-mediated knockdown of *GACAT3* suppresses the growth of HCT116 cells. Moreover, *GACAT3* promotes cell proliferation, colony formation, invasion, and migration in both HT29 and LoVo cells. *In vivo* studies have further confirmed the positive influence of *GACAT3* on CRC cell growth in a nude mice model (Zhou et al., 2018). Clinicopathological analyses of CRC suggest that *GACAT3* is positively related to tumor infiltration depth, TNM stage, lymph node metastasis, and CA19-9 level (Ye et al., 2020). Indeed, *GACAT3* expression in CRC patients with early (T1–2) disease is elevated compared to those with other T statuses. Likewise, *GACAT3* is upregulated in TNM stage III–IV tissues compared to stage I–II tissues. Together, these observations suggest that *GACAT3* distinguishes CRC from normal tissue and may also indicate tumor stage, supporting the development of *GACAT3* as a biomarker in CRC.

### Hepatocellular Carcinoma

Liver cancer is the second major cause of cancer-related death worldwide and accounts for approximately 850,000 new cancer cases annually (Llovet et al., 2016). Hepatocellular carcinoma (HCC) is the most common type of liver cancer (Anwanwan et al., 2020), and overexpression of many lncRNAs in hepatocytes contributes to carcinogenesis in HCC (Wang et al., 2017; Feng et al., 2019; Zhang et al., 2019; Zhang et al., 2020b). *GACAT3* is aberrantly overexpressed in HCC tissues and liver cancer cell lines, including HepG2, HCCLM3, SK-Hep-1, SMMC-7721, and Huh7 (Dong et al., 2020; Li et al., 2020). Importantly, *GACAT3* is associated with lower OS and disease-free survival in HCC patients (Dong et al., 2020). Studies in tissues and cell lines agree that inhibition of *GACAT3* suppresses the ability of hepatoma cells to proliferate and migrate and promotes cell apoptosis(Dong et al., 2020).

### Breast Cancer

Breast cancer is the most frequently diagnosed cancer and is the leading cause of cancer-related deaths among females (Winters et al., 2017). Most breast cancers are estrogen-receptor positive (70% of breast cancers), followed by triple negative breast cancers (15–20% of breast cancers), and finally by human epidermal growth factor 2 positive breast cancers (10–15% of breast cancers) (Yeo and Guan, 2017; Waks and Winer, 2019). The evolution of whole genome and transcriptome sequencing techniques has made lncRNAs essential for breast cancer research (Elias-Rizk et al., 2020; Oshi et al., 2020; Tsang and Tse, 2020). Studies have observed higher *GACAT3* expression in breast cancer tissues and MCF-7 cells compared to adjacent normal tissues and the normal breast epithelial MCF-10A cell line (Zhong et al., 2018; Hu et al., 2019b). Downregulation of *GACAT3* significantly inhibits proliferation, migration, and invasion of breast cancer cells.

Mammography is widely used to screen and diagnose breast cancer (Gøtzsche and Jørgensen, 2013; Løberg et al., 2015). Due to the high false-positive rate of mammography, recent studies have recommended breast MRI as a more sensitive assessment of the malignancy (McDonald et al., 2016). Notably, a study that employed breast MRI and diffusion-weighted imaging found that elevated expression of *GACAT3* was associated with reduced perfusion-related diffusion and increased perfusion fraction, which are indicative of aggressive tumor biology and undesirable chemotherapy effects (Hu et al., 2019b).

### Glioma

Glioma is the most common primary intracranial neoplasm in adults, accounting for 81% of brain tumors (Schwartzbaum et al., 2006; Jiang et al., 2016; Gusyatiner and Hegi, 2018). Although gliomas are relatively rare, they are aggressive tumors with high morbidity and mortality rates (Ostrom et al., 2014). LncRNAs are aberrantly expressed in glioma tissues and cell lines and are thought to affect the occurrence and development of the tumor (Peng et al., 2018; Yu et al., 2021). Recent studies have shown that *GACAT3* is overexpressed in glioma tissues and is incrementally elevated during disease progression. Based on these studies, *GACAT3* is included in the World Health Organization grading of gliomas. *GACAT3* expression is also substantially higher in the glioma cell lines U87, U251, and A172 compared to the normal brain cell line HA 1800. Experimental evidence suggests that *GACAT3* functions as an oncogene by regulating proliferation, apoptosis, migration, and invasion in glioma cells (Pan et al., 2019; Wang et al., 2019a).

### Bladder Cancer

Bladder cancer is one of the most common urological malignancies worldwide (Sung et al., 2021). The gold standard for bladder cancer diagnosis, urine cytology, is limited by low sensitivity (Ng et al., 2021). Emerging studies have shown that immune-related lncRNAs play fundamental roles in the prognosis and immunotherapy of bladder cancer patients (Wu et al., 2020; Zhou et al., 2021). *GACAT3* is upregulated in bladder cancer tissues, and elevated *GACAT3* expression positively correlates with advanced bladder cancer grade and stage. In CRISPR-Cas13-transfected bladder cancer T24 and 5637 cell lines, *GACAT3* promotes cell proliferation, suppresses apoptosis, and enhances cell migration by downregulating p21, BAX, and E-Cadherin protein levels, respectively. Mechanistically, *GACAT3* acts as a ceRNA by interacting with miRNA-497 in the cytoplasm (Zhang et al., 2020a). These observations indicate that *GACAT3*-targeted therapy is a promising strategy for bladder cancer.

### Non-Small Cell Lung Cancer

Lung cancer is the most frequent cancer worldwide (Hirsch et al., 2017) with an estimated 235,760 new cases and 131,880 mortalities in 2021. Lung cancer can be divided into small cell lung cancer (SCLC) and non-small cell lung cancer (NSCLC). The most common histologic subtypes of NSCLC are adenocarcinoma, squamous cell carcinoma, and large cell lung carcinoma (Kerr et al., 2014). Similar to major biomarkers of NSCLC, including epidermal growth factor receptor (EGFR), anaplastic lymphoma kinase (ALK), and Kirsten rat sarcoma viral oncogene homolog (KRAS) (Villalobos and Wistuba, 2017), *GACAT3* is upregulated in NSCLC tissues and cell lines. Clinicopathologic analyses indicate that *GACAT3* overexpression is significantly associated with lymph node metastasis and TNM stage but is not correlated with age, gender, or smoking history. Functionally, *GACAT3* overexpression promotes cell proliferation and migration in NSCLC (Yang et al., 2018). Of note, *GACAT3* enhances the sensitivity of lung cancer cells to radiotherapy, thereby promoting the apoptosis of NSCLC cells.

## Regulation of Tumorigenesis and Progression

Tumorigenesis occurs as a series of events that include proliferation, migration, and local invasion. Proliferation, migration, and invasion are the three primary features of malignant cells. *GACAT3* has been implicated in these three processes as well as in tumor growth, apoptosis, metastasis, epithelial-mesenchymal transition (EMT), and drug resistance. We discuss the importance of the relationship between *GACAT3* and tumor cell proliferation, apoptosis, migration, and invasion below. Additionally, we describe the molecular mechanisms underlying these biological processes, which are also summarized in Table 2.

**TABLE 2:** The function and related mechanisms of *GACAT3.*

Cancer type	Role	Function	Related mechanisms	References
Gastric cancer	Oncogene	Cell proliferation, colony formation, apoptosis, migration, and invasion	HMGA1, STAT3, BAX, miR-497, Ki-67, MMP-9, MMP-2, IL6, cyclin D, and miR-129-5p	Lin et al. (2018), Feng et al. (2018), Shen et al. (2016), Xu et al. (2014), Sun et al. (2016), Li et al. (2016), Xia et al. (2014), Song et al. (2013), Li et al. (2014)
Colorectal cancer	Oncogene	Cell proliferation, migration, and invasion	miR-103, LINC00152, miR-149, SP1, and STAT3	Ye et al. (2020), Zhou et al., 2018
Hepatocellular carcinoma	Oncogene	Cell proliferation, apoptosis, migration, invasion, and EMT	N-cadherin, *β*-catenin, TGF-*β*1, E-cadherin, BAX, and Bcl-2	Li et al. (2020), Dong et al. (2020)
Breast cancer	Oncogene	Cell proliferation, apoptosis, migration, and invasion	miR-497, caspase 9, Bcl-2, and CCND2	Hu et al., 2019b, Zhong et al. (2018)
Glioma	Oncogene	Cell proliferation, colony formation, apoptosis, migration, and invasion	miR-135a, NAMPT, miR-3127-5p, and ELAVL1	Wang et al., 2019a, pan et al., 2019
Bladder cancer	Oncogene	Cell proliferation, apoptosis, and migration	miR-497, p21, BAX, and E-cadherin	Zhang et al. (2021)
Non-small cell lung cancer	Oncogene	Cell proliferation and migration	TIMP2 and MMP10	Yang et al. (2018)

### Cell Growth and Proliferation

Tumor growth is tightly related to dysregulated cell proliferation. Unrestrained tumor cell proliferation, which is usually caused by the activation of oncogenes or inactivation of tumor suppressor genes, eventually results in tumor formation and progression (Kabała-Dzik et al., 2017; Zhang et al., 2021). Functional analyses from several tumor types suggest that *GACAT3* promotes cell proliferation and tumor growth by interacting with a variety of miRNAs and signaling pathways.

**FIGURE 1:**
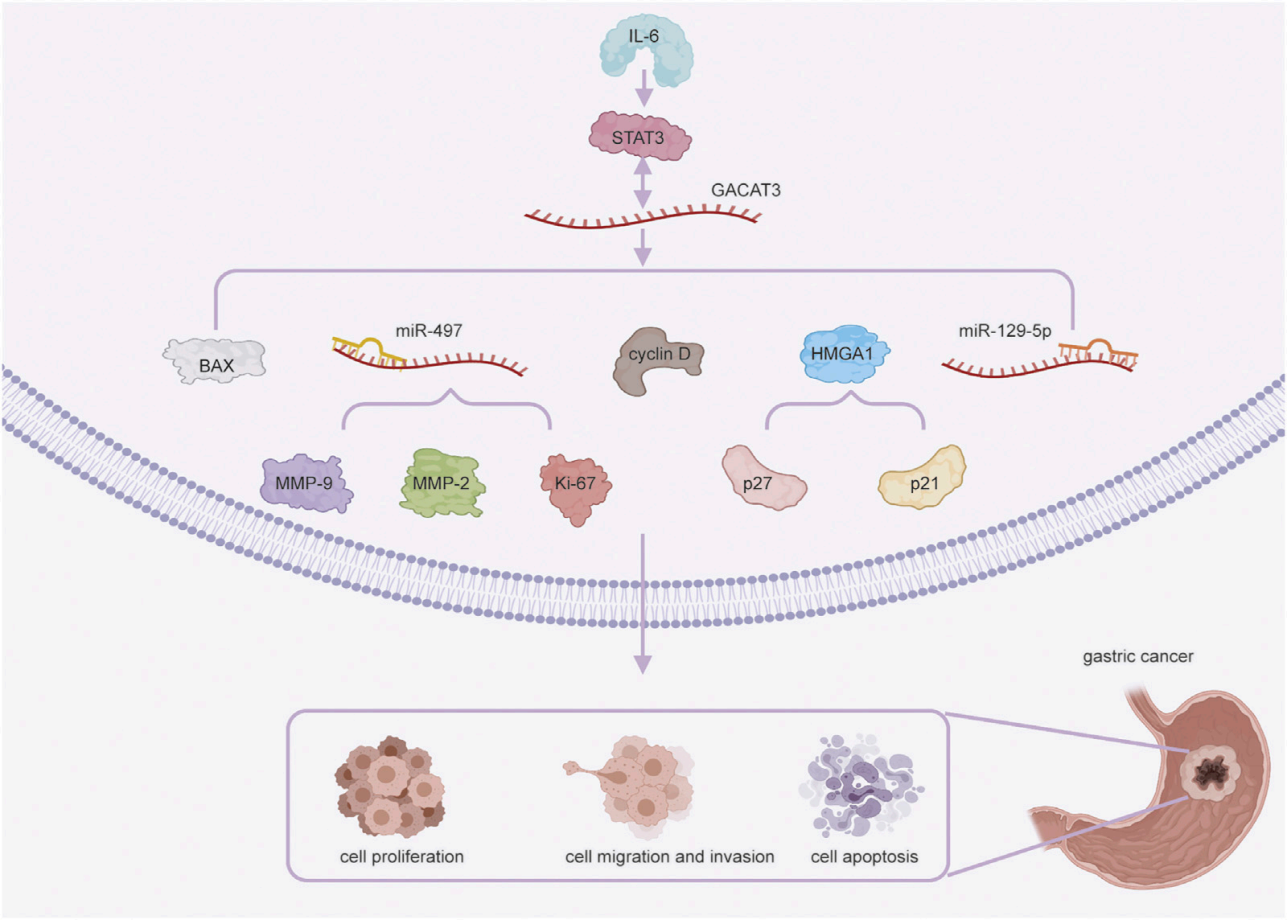
*GACAT3* in gastric cancer (GC) cell proliferation, migration, invasion, and apoptosis. *GACAT3* regulates cell proliferation in GC through multiple mechanisms. First, *GACAT3* competitively interacts with miR-129-5P. *GACAT3* also interacts with miR-497 to induce Ki-67 expression. Additionally, GACAT3 modulates the expression of HMGA1 and the levels of p27 and p21. Finally, *GACAT3* upregulates cyclin D expression *via* IL6/STAT3 signaling. *GACAT3* exerts its pro-migratory and pro-invasive effects by sponging miR-497 to downregulate the expression of MMP-2 and MMP-9. *GACAT3* promotes cell apoptosis by upregulating STAT3 expression and decreasing the level of BAX.

In GC, *GACAT3* regulates cell proliferation *via* multiple mechanisms (Figure 1). The interaction of *GACAT3* and miR-129-5P directly promotes proliferation (Xu et al., 2014), and the interaction of *GACAT3* and miR-497 induces expression of the proliferation marker Ki-67 in SGC-7901 cells (Feng et al., 2018). Additionally, *GACAT3* acts as a ceRNA of HMGA1, and this interaction suppresses cell cycle inhibitors p27 and p21 in GC SGC-7901 and BGC-823 cells. Finally, GACAT3 mediates cell proliferation downstream of the IL6/STAT3 signaling pathway by inducing cyclin D expression (Shen et al., 2016; Lin et al., 2018).

**FIGURE 2:**
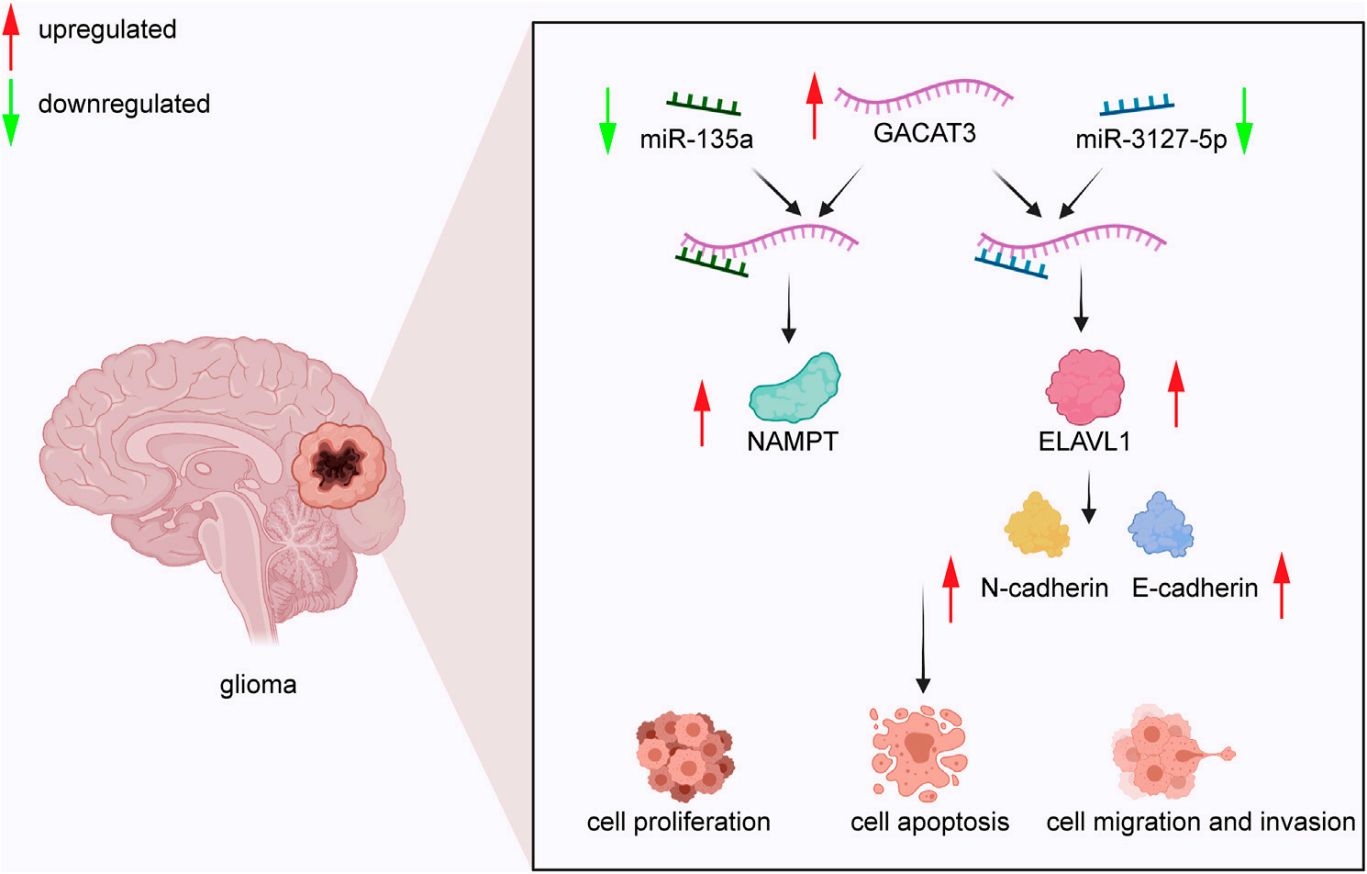
*GACAT3* in glioma. *GACAT3* elevates the level of NAMPT by interacting with miR-135a to mediate glioma cell proliferation. Additionally, *GACAT3* inhibits cell apoptosis by binding miR-3127-5p and consequently elevating ELAVL1 levels. Elevated ELAVL1 levels also enhance EMT by altering the balance of N-cadherin and E-cadherin. As a result, the *GACAT3*/miR-3127-5p/ELAVL1 pathway promotes cell migration and invasion.

In CRC, *GACAT3* stimulates cell proliferation *via* competitive interaction with miR-103 as the ceRNA of LINC00152 (Ye et al., 2020). In HCC, *GACAT3* facilitates cell proliferation in MHCC-97H, HepG2, and MHCC-LM3 cell lines; although, the mechanism remains unclear (Dong et al., 2020). In breast cancer, *GACAT3* promotes cell proliferation by reducing the levels of caspase 9 and upregulating the expression of Bcl-2 in MCF-7 cells. Alternatively, *GACAT3* can also regulate CCND2 expression by sponging miR-497 to enhance cell proliferation in MCF-7 cells (Zhong et al., 2018; Hu et al., 2019b). In glioma (Figure 2), *GACAT3* targets miR-3127-5p to downregulate ELAVL1 expression, thereby significantly enhancing cell proliferation and colony formation of A172 cells, U251 cells, and xenograft tumors (Pan et al., 2019). Moreover, *GACAT3* also mediates glioma cell proliferation by regulating NAMPT *via* competitive binding to miR-135a (Wang et al., 2019a). In NSCLC, *GACAT3* plays a positive role in tumor growth and cell proliferation by directly targeting TIMP2 in xenografts and A549 cells (Yang et al., 2018). Although *GACAT3* does not promote malignant cell proliferation *via* one consistent pathway or interaction, it does appear to influence proliferation *via* its ceRNA function in multiple tumor types, which may represent a therapeutic vulnerability.

### Cell Apoptosis

Apoptosis is a form of programmed cell death that maintains homeostasis in the body (Yue et al., 2020). Unsurprisingly, malignant cells often acquire the ability to evade apoptosis. Thus, to induce tumor cell death, many therapies target cell apoptosis or the pathways that allow tumor cells to evade apoptosis (Dong et al., 2015).

The anticancer drug cucurbitacin B promotes GC cell apoptosis, which is impaired by *GACAT3* expression. Mechanistically, *GACAT3* upregulates STAT3 to ultimately decrease expression of the pro-apoptotic protein bcl-2-associated X (BAX) in GC (Lin et al., 2018). In liver cancer cell lines, reduced expression of BAX and elevated expression of Bcl-2 (anti-apoptotic) support the anti-apoptotic effect of *GACAT3* (Dong et al., 2020). In breast cancer, *GACAT3* promotes cell apoptosis by downregulating caspases 3 and 9 as well as by upregulating BAX and Bcl-2 *via* miR-497 in MCF-7 cells (Zhong et al., 2018). In glioma, both *in vitro* and *in vivo* studies demonstrate that *GACAT3* inhibits apoptosis by attenuating miR-3127-5p and consequently elevating ELAVL1 levels (Pan et al., 2019). In general, *GACAT3* promotes the survival of malignant cells by impairing apoptosis and is a promising target to sensitive malignant cells to anti-cancer therapies.

### Cell Migration and Invasion

Metastasis is a hallmark of malignant tumors and a complicated process that involves cell adhesion, migration, and invasion (van Zijl et al., 2011). Due to advanced stage, recurrence, and distant metastasis, the overall 5-year survival rate of cancer patients remains poor (Borriello et al., 2021). Although EMT is commonly accepted as a key event in metastasis (Thiery et al., 2009; van Zijl et al., 2011), understanding the molecular mechanisms of tumor cell migration and invasion remains an important focus of cancer research.

In GC, the interaction of miR-497 and *GACAT3* influences cell migration and invasion by downregulating the cell invasion proteins MMP-2 and MMP-9 (Feng et al., 2018). *GACAT3* acts as an endogenous miRNA sponge for miR-149 in CRC, thereby enhancing cell migration and proliferation (Zhou et al., 2018). In liver cancer, the expression of EMT factors is altered following suppression of *GACAT3* in HepG2 and HCCLM3 cell lines. Specifically, N-cadherin, *β*-catenin, and TGF-*β*1 levels are reduced, and E-cadherin levels are elevated (Dong et al., 2020; Li et al., 2020), demonstrating that *GACAT3* has important implications for cell migration and invasion in liver cancer. In breast cancer, wounding healing assays in MCF-7 cells show that *GACAT3* knockdown dramatically decreases cell migration. Moreover, the expression of E-Cadherin was increased and the expression of N-Cadherin and Vimentin were decreased by *GACAT3* knockdown. Interestingly, these effects were suppressed by a miR-497 inhibitor, indicating that miR-497 and *GACAT3* cooperate to induce EMT and to enhance cell invasion and migration (Zhong et al., 2018). In glioma, *GACAT3* substantially upregulates NAMPT expression, which enhances cell migration and invasion *via* sponging miR-135a in U87 and U251 cells (Wang et al., 2019a; Cao et al., 2020). Another study reported that silencing *GACAT3* in U251 and A172 cells inhibited EMT by suppressing the levels of N-cadherin and elevating the levels of E-cadherin. Suppressed EMT further impairs cell migration and invasion *via* the *GACAT3*/miR-3127-5p/ELAVL1 pathway (Pan et al., 2019). In NSCLC A549 cells, *GACAT3* directly targets TIMP2 and upregulates MMP10 to foster cell invasion and migration (Yang et al., 2018). Together, these studies suggest that *GACAT3* contributes to cell invasion and migration, often by modulating the expression of EMT factors.

## Future Clinical Applications

Evidence from recent decades indicates that lncRNAs are vital regulators of human cancers (Xiao et al., 2021). As such, lncRNAs have great potential for clinical applications in multiple tumor types (Hu et al., 2019a; Lou et al., 2021). Based on the above discussion, *GACAT3* is a diagnostic and prognostic biomarker that has potential for next generation targeted therapies for specific tumors.

### Diagnostic Biomarker

Accurate and early diagnosis is critical for prolonging the survival of cancer patients (Wang et al., 2020). *GACAT3* is aberrantly expressed in GC, CRC, HCC, breast cancer, bladder cancer, and glioma. Abnormal expression of *GACAT3* in specific cancer tissues can reliably differentiate normal tissue from diseased tissue, suggesting that *GACAT3* is a useful diagnostic biomarker in these cancers. For example, ROC curve analysis validated the diagnostic value of *GACAT3* in CRC. The study demonstrated areas under ROC curve (AUCs) of 0.8183 and 0.6238 for *GACAT3* in 30 fresh CRC samples and 406 paraffin-embedded CRC samples, respectively. Co-detection of *GACAT3* and LINC00152 further improved diagnostic accuracy to AUCs of 0.8411 and 0.6675, respectively (Ye et al., 2020). *GACAT3* has also been confirmed as a diagnostic biomarker in glioma (Wang et al., 2019a) and is predictive of progression in breast cancer (Hu et al., 2019b).

### Prognostic Indicator

In addition to its potential as a diagnostic biomarker, *GACAT3* is also a promising prognostic indicator. Tumors discovered at advanced stages usually have a poor prognosis, which remains a major challenge for improving tumor outcomes. As tumor detection techniques are further developed, diverse molecular markers have become available to predict the prognosis of various tumors (Ma et al., 2020). *GACAT3* is widely thought to be a prognostic biomarker for multiple tumors. For example, *GACAT3* is associated with poor prognosis and shorter overall survival (OS) in GC (Xu et al., 2014; Feng et al., 2018). Moreover, *GACAT3* is an independent prognostic factor for both poor OS and disease-free survival in HCC patients. Similarly, *GACAT3* is associated with reduced OS and poor prognosis in breast cancer (Hu et al., 2019b). *GACAT3* has also been implicated in the prognosis of CRC (Ye et al., 2020), glioma, and NSCLC (Yang et al., 2018; Wang et al., 2019a). A summary of these clinical associations can be found in Table 1.

### Tumor Therapy Target

Despite decades of intensive cancer research efforts, there is no effective strategy to substantially decrease the rates of cancer recurrence and mortality (Guo et al., 2015; Song et al., 2018; Pienta et al., 2020). A recent breakthrough in cancer research has been the discovery and application of lncRNA in comprehensive tumor therapy (Bhan et al., 2017; Peng et al., 2017; Wang et al., 2019b). An abundance of evidence has demonstrated that *GACAT3* is a novel oncogenic lncRNA that functions as a ceRNA to modulate the occurrence and development of various tumors (Feng et al., 2018; Zhong et al., 2018; Zhang et al., 2020a).

Knockdown of *GACAT3* and overexpression of its relevant miRNA networks suppresses tumor progression, suggesting *GACAT3* is a potential therapeutic target (Shen et al., 2016; Zhou et al., 2018; Dong et al., 2020). Specifically, *GACAT3* is considered to be a valuable target for therapeutic intervention in GC, CRC, HCC, breast cancer, bladder cancer, NSCLC, and glioma (Pan et al., 2019). *GACAT3* weakens the anti-apoptotic effects of anticancer drugs in GC (Lin et al., 2018). Moreover, *GACAT3* is involved in the IL6 /STAT3 signaling pathway and therefore may be a promising biomarker for anti-cytokine treatment in GC (Shen et al., 2016). In NSCLC, overexpression of *GACAT3* enhances radiotherapy efficiency, providing a new therapeutic target for NSCLC (Yang et al., 2018). Moreover, *GACAT3* is included in an established ovarian cancer eight-lncRNA signature, which predicts chemotherapy response and platinum-resistance (Zhou et al., 2016; Klymenko et al., 2017). Despite these advances, the oncogenic mechanism of *GACAT3* remains vague. Additional in-depth exploration of *GACAT3* mechanisms are necessary to design effective cancer therapies.

## Conclusion and Future Outlook

Emerging evidence demonstrates that *GACAT3* is remarkably upregulated in CRC, HCC, breast cancer, NSCLC, bladder cancer, and glioma but shows diverse expression in GC cell lines. *GACAT3* expression levels in tumor samples are generally correlated with tumor size, metastasis to lymph nodes, TNM stages, and patient survival. Moreover, *GACAT3* is associated with CA19-9 levels in HCC and poor MRI diffusion weighted imaging in breast cancer, which are both indicative of aggressive tumors.

Functional analyses clearly demonstrate that *GACAT3* acts as a ceRNA and interacts with multiple miRNAs in relevant oncogenic signaling pathways. *GACAT3* exerts its carcinogenic effects by inhibiting apoptosis and enhancing cell proliferation, migration, and invasion. Thus, *GACAT3* is a potential tumor biomarker for cancer diagnosis, prognosis, and targeted therapy. Detection of *GACAT3* could be implemented to screen for malignancy and to monitor cancer progression. Specific *GACAT3*-targeted drug therapy offers a new direction and hope for cancer treatment. However, many of these findings were established in tissues and cancer cell lines. To fully explore the extent to which patients can benefit from *GACAT3* monitoring and targeted treatment strategies, additional clinical validation in cancer patients is necessary.
